# 165. Decreased Antimicrobial Consumption and Decreased Rates of Multi-drug Resistant Organisms Following Onset of the COVID-19 Pandemic: Experience from an Australian Tertiary Hospital

**DOI:** 10.1093/ofid/ofab466.367

**Published:** 2021-12-04

**Authors:** Michael Moso, Kelly Cairns, Trisha Peel, Nenad Macesic

**Affiliations:** 1 The Alfred and Central Clinical School, Monash University, Melbourne, Victoria, Australia; 2 Alfred Health, Melbourne, Victoria, Australia

## Abstract

**Background:**

Current guidelines recommend empiric antibiotics be used only for severe cases of coronavirus disease 2019 (COVID-19) or in cases where there is high clinical suspicion for bacterial co-infection. Level of adherence to guideline-recommended prescribing is unknown and high rates of antimicrobial prescribing may lead to increased development of resistance.

**Methods:**

We reviewed antimicrobial prescribing patterns for patients with COVID-19 managed at The Alfred Hospital in Melbourne, Australia in 2020. Adherence to World Health Organization (WHO) guideline-based prescribing was assessed by manual review of case notes. Monthly hospital-wide antibacterial consumption April-Dec 2020 (post-pandemic period) was compared to Jan 2019-Mar 2020 (pre-pandemic period), measured as days of therapy (DOT) per 1000 patient-days. Rates of multi-drug resistant organisms (MRO) (including MRSA, VRE, CPE, ESBL) were compared between months in 2019 and 2020 after pandemic onset (April 2020) and expressed as isolates per 1000 patient-days.

**Results:**

147 patients were managed for COVID-19 in 2020 at our centre. 101 patients required hospital admission and 58 (39%) were classified as either severe or critical in severity. 80 (54%) patients received empiric antimicrobial treatment, including 78/101 (77%) of hospital inpatients and 24/26 (92%) of ICU-admitted patients. 59 (73%) of antimicrobial prescriptions were adherent to WHO guidelines. Monthly antibacterial consumption was significantly lower post-pandemic than in the pre-pandemic period (mean 853 vs 902 DOT/1000 patient-days, *P=*0.0065). Antimicrobial use patterns varied, with significant decreases in commonly used antibiotics such as ceftriaxone, piperacillin-tazobactam, azithromycin and ciprofloxacin but no change in vancomycin or meropenem (Figure 1). There was a mean decrease of 0.77 MRO isolates/1000 patient-days (*P=*0.026) when each month in 2020 was compared with the corresponding month in 2019 (Figure 2).

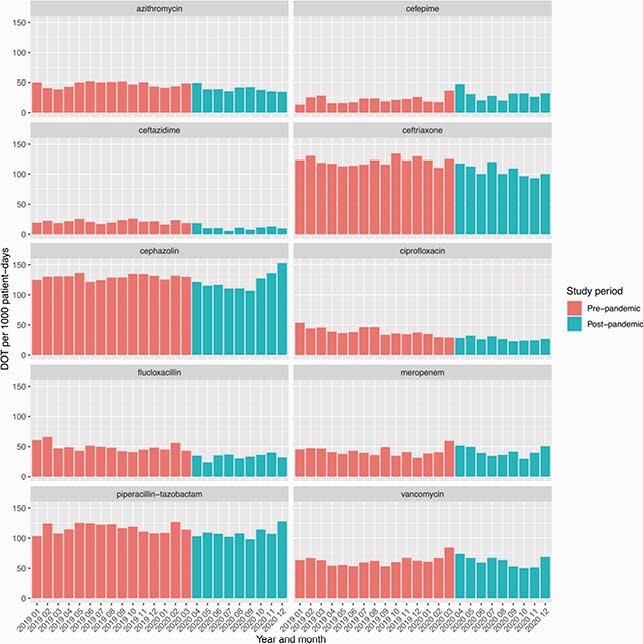

Antibacterial consumption in 2019 and 2020 by month, expressed as days of therapy/1000 patient-days.

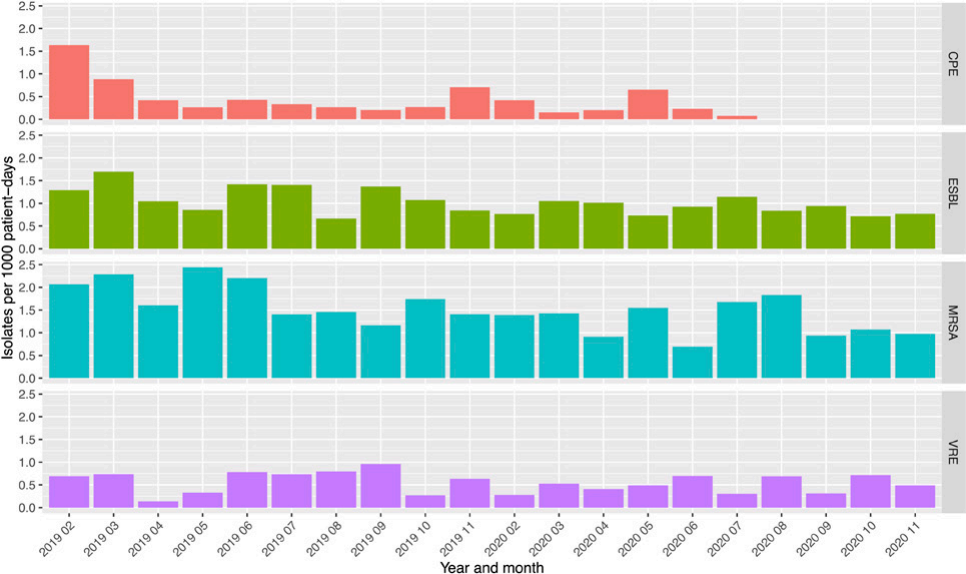

Rates of isolated multi-drug resistant organisms in 2019 and 2020 by month, expressed as isolates/1000 patient-days.

**Conclusion:**

A high proportion of admitted patients with COVID-19 received empiric antibiotics. In spite of this, we observed a significant reduction in total antimicrobial consumption and reduced rates of MRO isolation in the post-pandemic period.

**Disclosures:**

**All Authors**: No reported disclosures

